# RRFNet: A free-anchor brain tumor detection and classification network based on reparameterization technology

**DOI:** 10.1371/journal.pone.0325483

**Published:** 2025-06-16

**Authors:** Wei Liu, Xingxin Guo

**Affiliations:** School of Informatics, Hunan University of Chinese Medicine, Changsha, Hunan, China; Prince Mohammad Bin Fahd University, SAUDI ARABIA

## Abstract

Advancements in medical imaging technology have facilitated the acquisition of high-quality brain images through computed tomography (CT) or magnetic resonance imaging (MRI), enabling professional brain specialists to diagnose brain tumors more effectively. However, manual diagnosis is time-consuming, which has led to the growing importance of automatic detection and classification through brain imaging. Conventional object detection models for brain tumor detection face limitations in brain tumor detection owing to the significant differences between medical images and natural scene images, as well as challenges such as complex backgrounds, noise interference, and blurred boundaries between cancerous and normal tissues. This study investigates the application of deep learning to brain tumor detection, analyzing the effect of three factors, the number of model parameters, input data batch size, and the use of anchor boxes, on detection performance. Experimental results reveal that an excessive number of model parameters or the use of anchor boxes may reduce detection accuracy. However, increasing the number of brain tumor samples improves detection performance. This study, introduces a backbone network built using RepConv and RepC3, along with FGConcat feature map splicing module to optimize the brain tumor detection model. The experimental results show that the proposed RepConv-RepC3-FGConcat Network (RRFNet) can learn underlying semantic information about brain tumors during training stage, while maintaining a low number of parameters during inference, which improves the speed of brain tumor detection. Compared with YOLOv8, RRFNet achieved a higher accuracy in brain tumor detection, with a mAP value of 79.2%. This optimized approach enhances both accuracy and efficiency, which is essential in clinical settings where time and precision are critical.

## Introduction

A brain tumor is a collection or mass of abnormal cells in the brain. When a benign or malignant tumor grows within the confined space of the brain, it increases intracranial pressure, leading to brain damage and potentially life-threatening conditions. The brain is also a common site for metastasis from other types of cancer, such as lung, breast, skin (melanoma), colon, kidney, and thyroid. According to the National Brain Tumor Association, approximately 787,000 people in the United States are affected by brain tumors [1], making early detection and classification of brain tumors a crucial area of research in medical imaging. Detecting brain tumors early significantly improves patient survival rates and plays a critical role in selecting more effective treatment options [2]. However, manual detection of brain tumors is very challenging, requiring highly specialized radiologists and considerable time to process large amounts of magnetic resonance imaging (MRI) data [3]. The use of automated classification technologies for detecting and classifying brain tumors in MRI images has been extensively studied to address these challenges. For example, computer-aided detection (CAD) technologies, which offer high accuracy, are used for classifying brain tumors in MRI images and have proven to be highly reliable [4].

Many medical imaging techniques can be used to examine the structure of the brain, such as computed tomography (CT), positron emission tomography (PET), and magnetic resonance imaging (MRI). Compared with PET and CT, MRI is considered better [5] as it enables a detailed examination of brain anatomy without requiring patients movement or exposing them to ionizing radiation. It clearly shows the tumor area for further treatment. Therefore, MRI has promoted the comprehensive study of the human brain and enabled in-depth analysis of brain tumors [6]. With the development of science and technology, automated brain tumor detection technologies have emerged, combining artificial intelligence with clinical medicine. Brain tumor detection models based on MRI images enable physicians to make more informed medical decisions, predict tumor progression more accurately, and anticipate patient responses, thus providing invaluable benefits for improving patients’ quality of life and advancing medical science and technology [7].

Several machine learning (ML) techniques have been proposed to analyze MRI images of brain tumors. In addition, medical applications have demonstrated the potential of hybrid models and traditional deep learning models [8]. Deep learning algorithms excel at understanding subtle patterns and extracting relevant features from complex imaging data. These models can detect minute abnormalities and distinguish them from normal brain tissue. In this study, after examining the quantity of parameters in the brain tumor detection model, the batch size of input brain tumor images and the impact of anchor boxes on the brain tumor detection performance, an improved RRFNet brain tumor detection model based on YOLOv8 was proposed to realize more accurate brain tumor detection. RRFNet uses reparameterization technology to learn additional brain tumor characteristics during the training phase. In the inference stage, only one convolution layer is retained through reparameterization, which accelerates the model’s inference speed.

The contributions of this paper can be summarized as follows.

(1) Reparameterization technology was employed to construct the backbone network of the brain tumor detection model with RepConv and RepC3 to extract richer semantic information of the brain tumor and ensure the detection speed of brain tumor detection model.

(2) The FGConcat module was introduced to achieve finer grained fusion of brain tumor feature maps, while the class activation mapping technique was used to visualize the model’s attention on the FGConcat module’s output.

(3) The RRFNet network model was proposed to improve brain tumor detection. RRFNet built the backbone network with RepConv and RepC3 and used FGConcat for brain tumor feature map fusion to realize more accurate brain tumor detection and recognition. The experimental results show that RRFNet has a mAP value of 79.2% on the brain tumor dataset, which is 0.9% higher than that of the YOLOv8 model.

This paper analyzes the effects of deep learning network parameter quantity, model depth, and use of anchor boxes on the performance of brain tumor detection models. It provides valuable insights for future research on brain tumor detection.

## Related work

In recent years, advancements in computer vision and machine learning related technologies have introduced new opportunities for the detection and classification of brain tumors. Modern approaches have increasingly automated brain tumor detection and classification through data-driven methods, leading to significant improvements in the efficiency and accuracy of brain tumor recognition tasks [9]. The development of automated brain tumor detection and classification technology can be divided into two key phases: the machine learning phase, which relies on manual feature extraction, and the phase utilizing deep learning technology.

### Machine learning object detection

In the manual feature extraction stage, image preprocessing is performed first, followed by manual feature extraction. This process involves exploring various aspects such as edge detection, texture features and shape features [10], requiring extensive experimentation and a deep understanding of both the domain and dataset to select suitable features or feature combinations for classification. Moreover, multiple experiments are necessary to identify the appropriate features for the dataset and to select the appropriate classifier algorithm. Additionally, designing features and selecting classifiers to get the best results is an extremely challenging and time-consuming task. Despite these challenges, studies have managed to overcome various obstacles, advancing automated brain tumor detection and classification tasks and achieving commendable results on brain tumor datasets [11].

Islam *et al*. [12] proposed a method based on the multiple Brownian motion (mBm) model to describe and segment brain tumor textures in MRI images. Additionally, they enhanced the AdaBoost algorithm to improve the classification of difficult samples. Experimental results demonstrate that the proposed method is highly effective and accurate for automatic segmentation of brain tumors, outperforming other existing techniques. Amin *et al*. [10], introduced a tumor segmentation method based on unsupervised clustering, utilizing a fusion feature vector that combines Gabor wavelet features (GWF), histograms of oriented gradient (HOG), local binary patterns (LBP), and segmentation-based fractal texture analysis (SFTA) features. A random forest (RF) classifier was employed to classify complete tumors, enhanced tumors, and non-enhanced tumors among three sub-tumor regions, improving classification accuracy between tumor regions. Manogaran *et al*. [13] proposed an improved machine learning method based on the orthogonal gamma distribution, which analyzes under- and over-segments of brain tumor regions to automatically detect anomalies in ROI, achieving accurate detection and segmentation of brain tumor regions. However, manual intervention methods are insufficient for accurately detecting and classifying tumor types. Although machine learning-based automatic detection technologies improve efficiency, their development process is lengthy because they require experts to preprocess images and extract manual features. Moreover, the accuracy of brain tumor classification largely depends on the features extracted by experts. Zacharaki *et al*. [14] employed several features, including shape, rotation-invariant texture, and intensity features, to classify tumor types. However, this feature selection strategy increases the risk of false positives, reducing classification accuracy. The model proposed in this literature only achieved classification accuracies between 79% and 85%. To address the limitations of manual feature extraction, some studies have shifted toward using deep neural networks for automatic image classification [15]. For example, Singh *et al*. in [16] used algorithms such as the naive Bayes classifier, K-nearest neighbor, and random forest to classify brain tumors; however, these methods achieved accuracy rates between 88% and 93%, which are lower than those achieved by deep learning methods.

### Deep learning object detection

With the advancement of technology, the traditional approach of manual feature extraction, followed by iterative generation of candidate boxes and final classification using support vector machine (SVM), presents several drawbacks, including low detection accuracy, long processing times, and the need for manual design of feature selectors. As technology has progressed, more advanced deep learning network models with superior detection capabilities have emerged [17, 18].

Bhanothu *et al*. [19] utilized the Faster R-CNN deep learning algorithm for tumor detection, marking regions of interest through a region proposal network (RPN). The MR image dataset used in the study included three types of primary brain tumors: gliomas, meningiomas, and pituitary tumors. The VGG-16 architecture was employed as the backbone network for both the RPN and the classifier. The detection and classification results showed an average accuracy of 75.18% for gliomas, 89.45% for meningiomas, and 68.18% for pituitary tumors, with the algorithm achieving an average precision of 77.60% across all categories. Dipu *et al*. [20] tested YOLOv5 and the deep learning library FastAi on a subset of the BRATS 2018 dataset. YOLOv5 achieved an accuracy rate of 85.95%, whereas FastAi reached an accuracy rate of 95.78%. Maqsood *et al*. [21] proposed a modified MobileNetV2 architecture for feature extraction and trained the model using transfer learning. They then employed an M-SVM for brain tumor classification to identify meningiomas, gliomas, and pituitary tumors. The method was evaluated on the BRATS 2018 and Figshare datasets, achieving an accuracy rate of 97.47% and 98.92%, respectively. To better detect the brain tumor area, Hossain *et al*. [22] conducted a performance study of various deep learning (DL) architectures to achieve faster, more reliable brain tumor detection. The architectures tested included Visual Geometry Group 16 (VGG16), InceptionV3, VGG19, ResNet50, InceptionResNetV2, and Xception. Based on the top three performing transfer learning (TL) models, a new multi-class classification model called IVX16 was proposed, which showed significant performance improvements in brain tumor detection and classification. To address the challenge of requiring a large number of manually annotated images in supervised learning, and the limitations of generative models in unsupervised methods, Wang *et al*. [23] introduced a novel two-stage generative model (TSGM) framework. This framework combines CycleGAN with variance exploding stochastic differential equations (VE-JP) using joint probability to improve brain tumor detection and segmentation. TSGM was validated on the BraTs2020, ITCS, and In-house datasets, demonstrating improved segmentation performance and strong generalization capabilities. Mecheter *et al*. proposed a new method that combines multi-resolution handcrafted features with features based on convolutional neural networks (CNNs) for the segmentation of MRI [24]. The experimental results showed promising results owing to the integration of multi-resolution and CNN features. To address issues such as loss of boundary information and incorrect regional classification, Liu *et al*. [25] introduced a spatial pyramid (SP) block module and attention mechanism. A new lightweight, scalable Swin Transformer network called IMS2Trans was proposed by Zhang *et al*. [26] for the segmentation of brain tumors with incomplete MRI modalities. This method enables efficient information sharing and fusion without sacrificing segmentation performance even in the absence of modalities.

### Summary

In the field of brain tumor detection, while each machine learning or deep learning model discussed above has achieved significant results and provided valuable references for subsequent research, they also have inherent limitations and shortcomings. Traditional machine learning algorithms require manual design of feature extractors, which is time-consuming and requires professional knowledge of brain tumors. Some deep learning models , particularly those with a large number of parameters, require substantial computational resources for training and inference, which may limit their application in resource-constrained environments. Moreover, as model complexity increases, interpretability becomes more challenging. In the medical field, the interpretability of models is crucial, as it helps doctors and researchers understand the decision-making process behind the model’s predictions. Furthermore, specific experimental research on the use of anchor boxes for brain tumor detection and classification is lacking.

Therefore, this study uses deep learning technology to analyze the performance of brain tumor detection models under the influence of factors, such as parameter volumes, input batch sizes, and anchor box strategies through experimentation. The aim is to explore the effect of these factors on model accuracy and efficiency, providing valuable insights for improving brain tumor detection. Additionally, this study visualizes the attention heat maps generated during the brain tumor detection process, further aiding in the understanding of the model’s decision-making mechanism.

## Methodology

### Dataset

**Fig 1:**
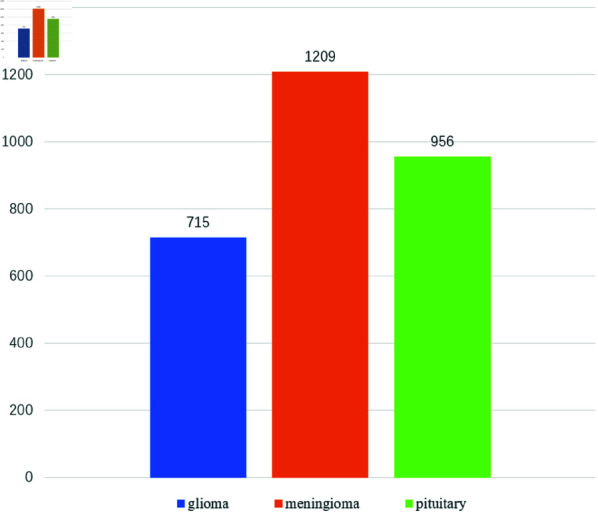
Brain tumor category distribution of training set.

**Fig 2:**
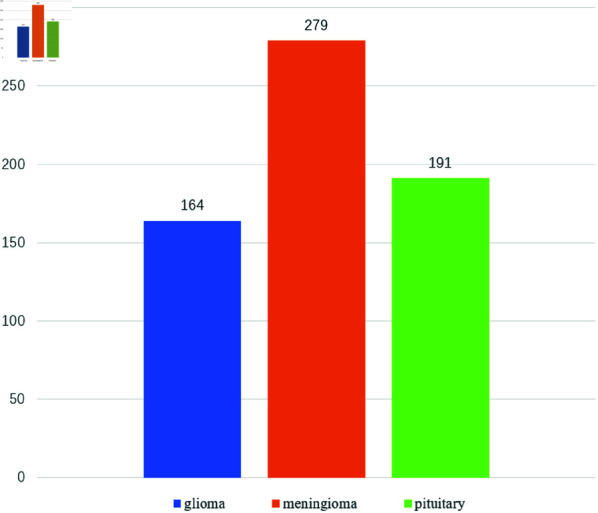
Brain tumor category distribution of test set.

The Brain Tumor MRI Dataset (BTMD) [27] consists of the Figshare, SARTAJ, and Br35H brain tumor datasets. It includes MRI images with four types of labels: glioma, meningioma, pituitary, and no tumor. The MRI provided in BTMD are a combination of T1, T2 and FLAIR types. As for the Figshare datasets, this brain tumor dataset consists T1-weighted contrast-enhanced images with three kinds of brain tumor. All images in Figshare dataset (T1-weighted contrast-enhanced MRI) were acquired after Gd-DTPA injection at Nanfang Hospital, Guangzhou, China and General Hospital, Tianjin Medical University, China from 2005.9 to 2010.10. The images have an in-plane resolution of 512 × 512 with pixel dimensions of $0.49\times 0.49\;{\text{mm}}^{2}$ . The slice thickness is 6 mm and the slice gap is 1 mm. The Gd dose was 0.1 mmol/kg at a rate of 2 ml/s. However, neither SARTAJ nor Br35H provides relevant imaging information. During the preprocessing of the raw data, we filtered out images without brain tumors and those that were difficult to annotate. Subsequently, we used labelme to annotate the images to clearly identify the exact locations of the brain tumors. In total, we annotated 3,514 images. Through manual annotation, we divided 3,514 MRI brain tumor images into training and validation sets in an 8:2 ratio. The distribution of brain tumor categories in the training and test sets are shown in Figs 1 and 2, respectively.

**Fig 3:**
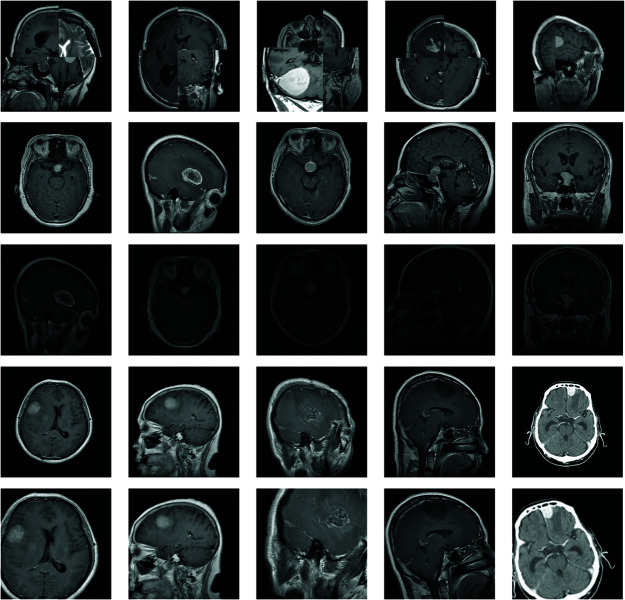
The first row presents brain tumor images augmented using the Mosaic technique, which randomly stitches four brain tumor images together to form a new composite image. The second row of brain tumor images is transformed into the third row through HSV data augmentation operations. The fourth row of brain tumor images is processed with translation, scaling, and rotation to produce the final row of brain tumor images.

To avoid class imbalance in the dataset, we ensured that the number of each brain tumor type in the training and validation sets did not exceed twice the number of other categories. As the number of brain tumor samples was still relatively small, we employed data augmentation techniques during the model training process. Data augmentation generates additional training data by creating modified versions of the original images [28]. This ensures that the model encounters diverse samples during training, thereby increasing dataset diversity. The technique is particularly useful for deep learning models when dealing with relatively small image datasets. This study utilized data augmentation techniques, such as rescaling, rotation, translation, cropping, scaling [29], as well as mosaic enhancement, to reduce overfitting and enhance the model’s generalization ability. The mosaic data augmentation technique, inspired by the CutMix [30] method, enriches the background for brain tumor detection by randomly cropping and stitching together four images to form a new image. This approach helps improve the model’s ability to recognize brain tumors in complex backgrounds. In the data augmentation phase, we meticulously configured the following parameters to enhance the robustness and generalization capability of our model. Specifically, the HSV color space augmentation was finely tuned with the hue fraction set to 0.015, saturation fraction to 0.7, and value fraction to 0.4. These settings allowed for subtle yet effective adjustments to the color characteristics of the images, thereby simulating diverse lighting and color conditions. Additionally, spatial augmentations were applied, including a translation fraction of 0.1 to introduce positional variability and a scaling gain of 0.5 to account for size variations in the objects of interest. Furthermore, a left-right flipping probability of 0.5 was implemented to ensure the model’s invariance to horizontal orientation changes. To more intuitively illustrate the effect of data augmentation, we have provided example images after data augmentation, as shown in Fig 3.

### Anchor boxes

**Fig 4:**
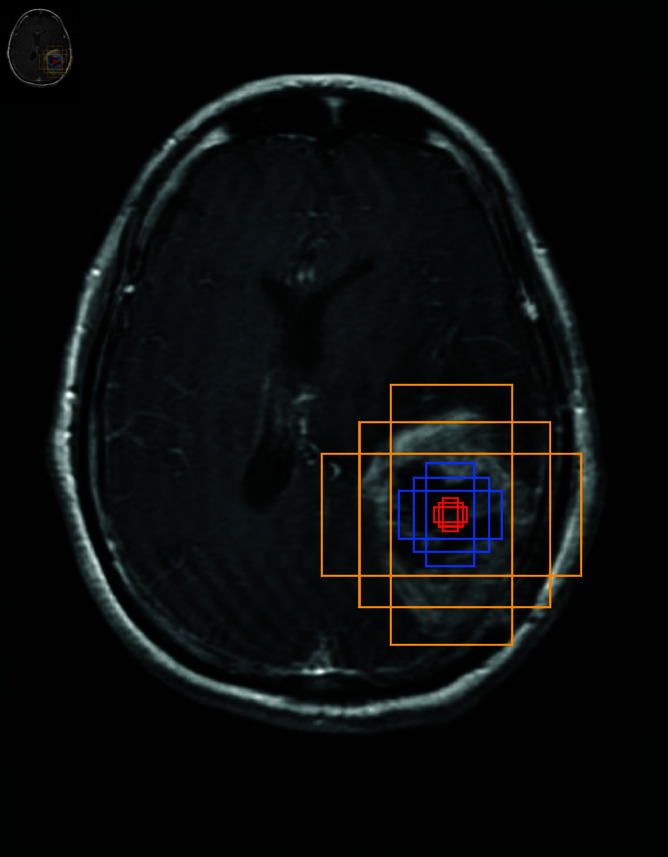
Three scales of anchor boxes settings, corresponding to yellow, blue and red three colors.

Anchor boxes are a crucial concept in object detection models. They are predefined bounding boxes used to predict the position and size of objects in detection tasks. Anchor boxes with fixed sizes and aspect ratios are placed on feature maps of images. The models then learn to adjust these boxes to match the true bounding boxes of objects. For instance, in the SSD algorithm, several prior boxes with different scales and aspect ratios are set at the same location to cover objects of different sizes shown in Fig 4. The original image is processed through a convolutional network to generate a feature map, and multiple anchor boxes of various sizes and aspect ratios are generated on each cell of the feature map to reduce computational load. The scale of an anchor box refers to its size, whereas the ratio describes its shape information, specifically the width-to-height proportion. During training, anchor boxes serve as training samples, requiring two labels for each box: a category label (the class of the object within the anchor box) and an offset (the displacement of the true bounding box relative to the anchor box). In the prediction phase, the model outputs category probabilities and bounding box offsets for each anchor box. In the post-processing stage, redundant predicted bounding boxes are removed using techniques such as non-maximum suppression (NMS), retaining the final prediction results. The design of anchor boxes can proceed in three directions: based on human experience, K-means clustering, or learning as hyperparameters. Anchor boxes have been implemented in various object detection models, such as Faster R-CNN, SSD, YOLOv2, YOLOv3, each enhancing object detection performance in different ways.

However, anchor box-based object detection methods rely on predefined scales and shapes, which increases the number of hyperparameters that need to be adjusted. In contrast, object detection models that do not rely on anchor boxes may exhibit improved generalization when objects of different shapes and sizes are present because they are not limited by the predefined anchor box structure. Moreover, as they simplify the model architecture and reduce computational steps, non-anchor-based models are more suitable for real-time object detection systems.

Certain models, such as YOLOv8 and DETR, directly predict bounding boxes on the feature map without the need for anchor boxes. DETR treats object detection as a set prediction problem, streamlining the detection process and eliminating the need for manually designed components, such as anchor box generation or the NMS. As they do not depend on predefined anchor box scales and shapes, they tend to achieve better results across more datasets and exhibit stronger generalization. To explore the impact of using or not using anchor boxes on the performance of brain tumor detection tasks, this study conducts comparative experiments, analyzing the differences between anchor-based and anchor-free YOLOv5 in brain tumor detection and recognition.

**Fig 5:**
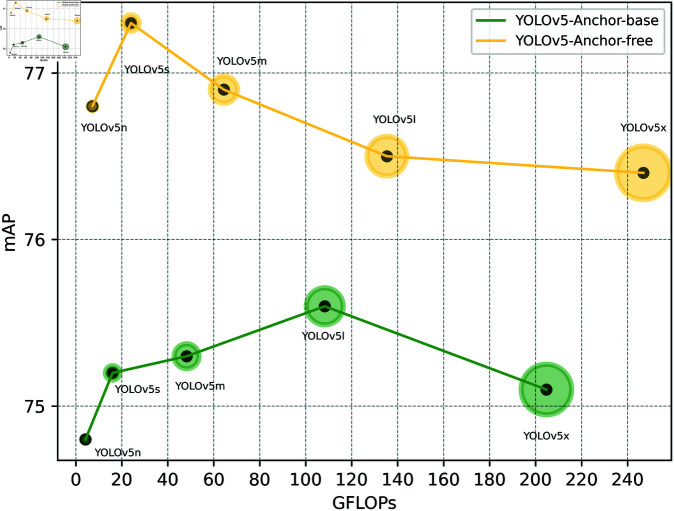
The line chart comparing the mAP of YOLOv5-Anchor-based and YOLOv5-Anchor-free on the brain tumor dataset.

Table 1 indicates that the anchor-free method achieves superior performance on the brain tumor dataset used in this study, with a nearly 2 mAP difference between the two models. Fig 5 illustrates a comparison between anchor-based and anchor-free brain tumor detection models. Evidently, the RRFNet brain tumor detection model proposed in this study presents improvements to the anchor-free brain tumor detection methods.

**Table 1:** The test results of YOLOv5 on the brain tumor dataset regarding whether it uses anchor-based detection.

Model	Params(M)	GFLOPs	Precision	Recall	AP50	mAP	FPS
YOLOv5n-anchor-free	2.5	7.2	96.2±0.2	94.1±0.1	97.1±0.1	76.8±0.1	91.8±2.2
YOLOv5s-anchor-free	9.1	24.0	96.2±0.2	93.6±0.3	97.2±0.1	77.3±0.2	69.9±0.5
YOLOv5m-anchor-free	25.1	64.4	95.8±0.1	93.5±0.5	97.3±0.2	76.9±0.1	58.7±2.1
YOLOv5l-anchor-free	53.2	135.3	96.1±0.5	93.7±0.1	96.6±0.3	76.5±0.1	38.5±0.5
YOLOv5x-anchor-free	97.2	246.9	96.3±0.3	92.8±0.3	96.5±0.1	76.4±0.1	21.9±0.3
YOLOv5n-anchor-base	1.8	4.2	95.2±0.5	93.3±0.2	95.2±0.2	74.8±0.2	145.1±3.1
YOLOv5s-anchor-base	7.0	16.0	95.2±0.3	93.1±0.4	94.9±0.2	75.2±0.1	126.1±4.8
YOLOv5m-anchor-base	20.9	48.2	95.8±0.4	93.3±0.2	95.7±0.3	75.3±0.2	91.5±5.5
YOLOv5l-anchor-base	46.1	108.3	95.3±0.3	93.4±0.1	95.9±0.2	75.6±0.2	54.2±0.3
YOLOv5x-anchor-base	86.2	204.7	95.6±0.2	93.0±0.2	95.2±0.3	75.1±0.2	30.6±0.5

### Batch size

This section discusses the comparative experiments performed on the models using different batch sizes. Batch size refers to the number of samples input into the model for training during each iteration. Various factors influence batch size, which significantly impacts model training and experimental results. A larger batch size provides a more accurate gradient estimate by averaging the loss function over multiple samples. Additionally, because the gradient updated at each iteration is more stable with a larger batch size, the model typically converges faster. However, an excessively large batch size can cause the model to fall into a local minimum rather than reaching the global minimum. From an implementation perspective, when using batch normalization (BN) to normalize the inputs of network layers, a larger batch size offers more stable BN operations. BN normalizes inputs by calculating the mean and variance based on the batch; therefore, a smaller batch size may lead to noisier estimates of mean and variance, which can affect network stability and performance. The mean and standard deviation of a mini-batch can be expressed as follows.

$$\mu_{B}=\frac{1}{m}\sum_{i=1}^{m}x_{i}$$ (1)

$$\sigma_{B}=\sqrt{\frac{1}{m}\sum_{i=1}^{m}{\left(x_{i}-\mu_{B}\right)}^{2}}$$ (2)

where $\mu_{B}$ represents the mean, $\sigma_{B}$ represents the standard deviation, and $B=\{x_{1},\ldots ,x_{m}\}$ represents the sample in the mini-batch.

$$BN(x)=\gamma ⊙\frac{x-{\hat{\mu }}_{B}}{{\hat{\sigma }}_{B}}+\beta$$ (3)

where γ,β are two learnable parameters, representing the scale and shift parameters, respectively.

### Proposed approach

**Fig 6:**
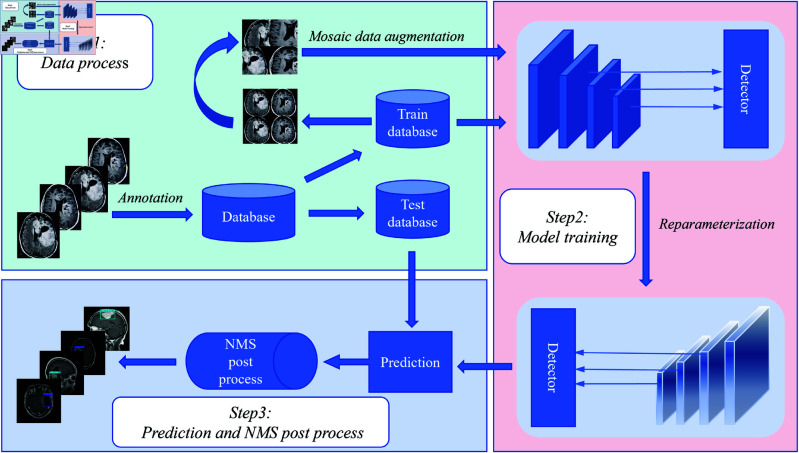
The overall diagram of the brain tumor detection process. The data processing stage includes brain tumor data annotation, division of training and testing sets, and data augmentation. After the brain tumor detection model is trained, it is reparameterized to obtain a lightweight model, which improves the model’s inference speed. In the post-processing stage, NMS is used to further enhance the quality of brain tumor detection results.

After exploring the impact of Anchor boxes and Batch size on tumor detection, this paper proposes a novel anchor-free object detection algorithm for tumors. The overall process is shown in the Fig 6. First, the tumor dataset is annotated and divided into a training set and a test set in a ratio of 8:2. During the training of the brain tumor training set, a series of data augmentation operations, such as Mosaic data augmentation, are used to increase the sample size of brain tumors and enhance the robustness of the brain tumor detection model. In the inference stage, the well-trained tumor detection model is reparameterized to integrate knowledge, reducing the model’s parameter volume to improve the detection speed of brain tumors. In the post-processing stage, NMS is used to eliminate candidate boxes with excessive IOU thresholds, achieving better detection results for brain tumors. The following sections will detail the core components of the brain tumor detection model, FGConcat, RepConv, RepC3, and the proposed RRFNet. The codes and data used in this study are available at https://github.com/Mr-cream/RRFNet.

### FGConcat

In deep learning models, feature maps are the multidimensional information learned from the input data, capturing various levels of features from the input images. The concatenation of feature maps is a widely used technique that enables models to effectively combine and integrate information across different network layers. To better capture multi-level feature information, this study introduces the finer-grained concat (FGConcat) module, which enables the model to enhance its expressive power while preserving spatial resolution. The FGConcat module starts by adjusting two input feature maps to the same channel dimension through a 1×1 convolutional layer; it then performs feature adjustment using a shared-parameter EMA attention mechanism [31], and finally fuses the optimized feature maps through concatenation. Assume there are two input feature maps, denoted as *M*_1_ and *M*_2_, with dimensions (*H*,*W*,*C*_1_) and (*H*,*W*,*C*_2_) respectively, where *H* and *W* represent the height and width of the feature maps, and *C*_1_ and *C*_2_ are the number of input channels.

$$M_{1}^{top}={\text{Conv}}_{3\times 3}({\text{Conv}}_{1\times 1}(M_{1}))+B_{1}^{top}$$ (4)

$$M^{mid}=\text{Concat}(M_{1},M_{2})$$ (5)

$$M_{2}^{bottom}={\text{Conv}}_{3\times 3}({\text{Conv}}_{1\times 1}(M_{2}))+B_{2}^{bottom}$$ (6)

where ${\text{Conv}}_{1\times 1}$ adjusts the number of channels of *M*_1_ and *M*_2_ from *C*_1_ and *C*_2_ to $C_{1}\;+\;C_{2}$ respectively. Finally, the feature maps are fused by element-wise addition through an Add operation.

$$M^{output}=\text{Add}(EMA(M_{1}^{top}),M^{mid},EMA(M_{2}^{bottom}))$$ (7)

The pseudocode implementation of the proposed FGConcat module is shown in Algorithm 1.

**Algorithm 1. FGConcat module PyTorch pseudocode.**

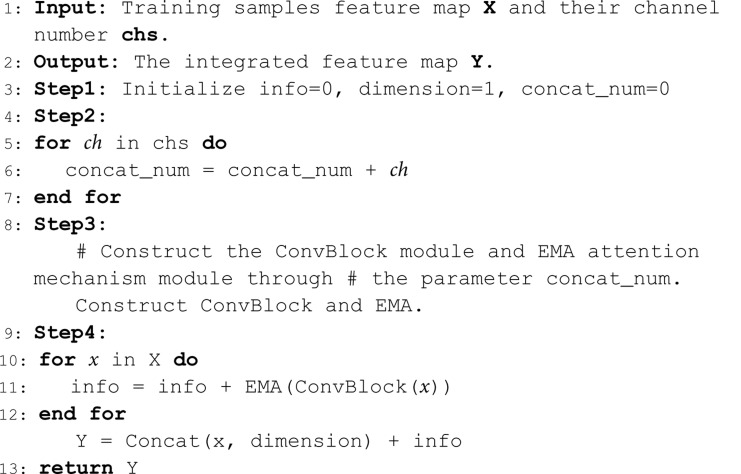



**Fig 7:**
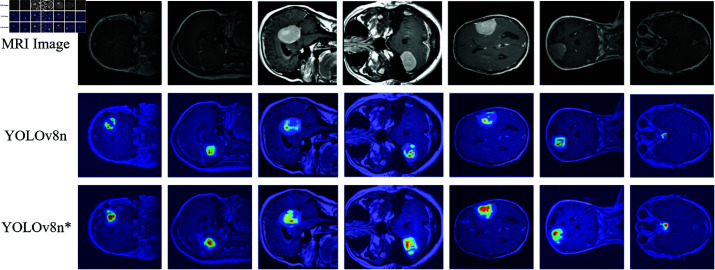
Heatmap comparison results, where column 3 (YOLOv8*) shows the experimental results of feature map fusion using FGConcat.

This study employs YOLOv8 as the baseline network, replacing the Concat module with the FGConcat module for visualization experiments. Class activation mapping technology [32] is used to visualize the output of the feature map concatenation layer in the form of heatmaps shown in Fig 7, highlighting the focus areas of the brain tumor detection model.

### RepConv and RepC3

**Fig 8:**
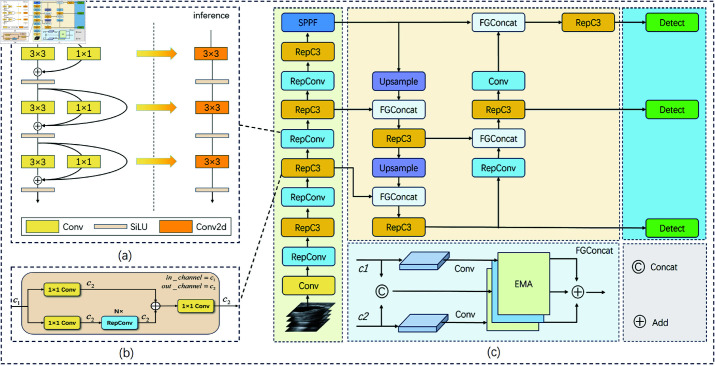
Overview of the proposed RRFNet. Area (a) represents the RepConv module, which employs reparameterization to reduce the computational load during model inference. Area (b) is the RepC3 module, which uses a dual-branch strategy to extract semantic information of brain tumors. Area (c) is the proposed FGConcat module, designed for fine-grained feature map fusion.

To enhance brain tumor detection performance, this study employs reparameterization technique to improve the model’s detection capabilities. RepVGG [33] is a convolutional neural network architecture that utilizes structural reparameterization, adopting a multi-branch topology during training but presenting a simple, single-path architecture composed of stacked 3×3 convolutional layers and ReLU activation functions during inference. This approach enables RepVGG to maintain the simplicity of VGG-style architecture while achieving performance comparable to more complex CNN architectures. RepConv, inspired by the coding style of RepVGG, modifies the original activation functions and other parts, resulting in performance improvement. The schematic diagram of training and inference for the network composed of RepConv is shown in Fig 8(a).

**Fig 9:**
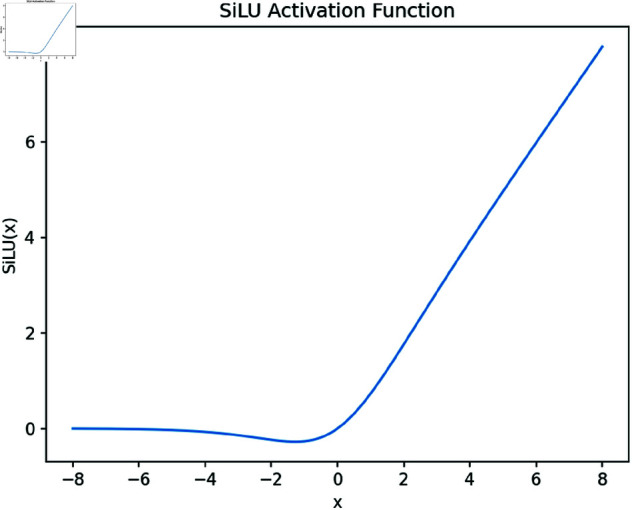
SiLU activation function diagram.

Activation functions and their derivatives (gradients) play a crucial role in the backpropagation process. During backpropagation, gradients are used to update the network’s weights and biases. Choosing the right activation function can help the network converge faster and reduce issues that may arise during training, such as gradient vanishing or explosion. RepConv uses sigmoid linear unit (SiLU) as the activation function, which, compared to ReLU, has self normalizing properties that helps reduce the problem of gradient vanishing in the training process and effectively improves the model’s convergence speed. The SiLU activation function diagram is shown in Fig 9.

RepC3 [34] is a bottleneck layer built around the core of RepConv. The bottleneck layer is an important component introduced in deep residual networks, such as ResNet, to reduce the computational complexity of the model and enhance feature extraction capabilities. Similar to the C3 module [35], the RepC3 module employs a 1×1 convolutional layer to adjust the number of input channels, while a 3×3 convolutional kernel in RepConv strengthens the feature representation of brain tumors. The module diagram of RepC3 is shown in Fig 8(b). Assume the input feature map *X* has dimensions (*H*,*W*,*C*_1_), where *H* and *W* represent the height and width of the feature map, respectively, and *C*_1_ denotes the number of input channels. The first convolution operation transforms the number of input channels from *C*_1_ to *C*_2_.

$$F_{1}={\text{Conv}}_{1\times 1}(X)+B_{1},F_{2}={\text{Conv}}_{1\times 1}(X)+B_{2}$$ (8)

where *X* is the input feature map, ${\text{Conv}}_{1\times 1}$ represents the 1×1 convolution operation, *B*_1_ and *B*_2_ are the bias. Subsequently, we feed *F*_2_ into the RepConv module, which is repeated *N* times (*N* = 3 in this paper).

$$F_{3}={\text{RepConv}}_{N}(F_{2})+B_{3}$$ (9)

Finally, we fuse the tumor features represented by *F*_1_ and *F*_3_ respectively.

$$F_{4}={\text{Conv}}_{1\times 1}(Concat(F1,F3))+B_{4}$$ (10)

### RRFNet

**Fig 10:**
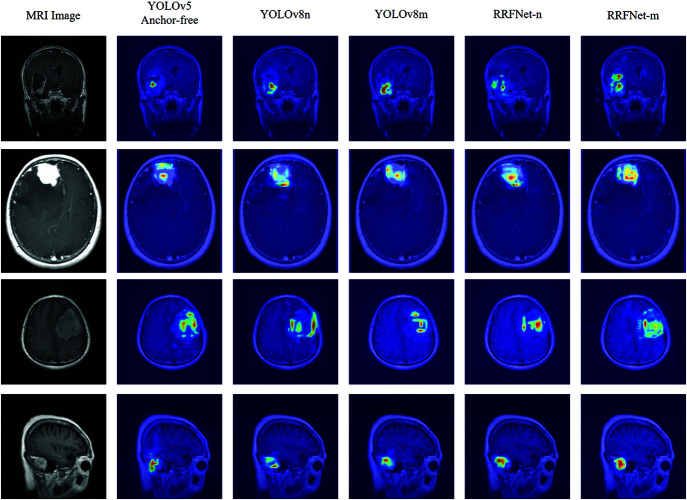
Comparison results of heatmap of different brain tumor detection models.

The architecture of RRFNet is illustrated in Fig 8. In the realm of anchor-free object detection models, YOLOv8 has demonstrated outstanding performance across a variety of detection tasks. Building upon YOLOv8, this study integrates RepConv and RepC3 into the backbone network to capture more comprehensive semantic information about brain tumors. For brain tumors that vary in scale, detections are made on feature maps of different scales through dedicated detection heads. Specifically, this study employs feature maps at scales of 20×20, 40×40, and 80×80. Larger brain tumors are detected on the 20×20 feature maps, medium-sized tumors on the 40×40 maps, and smaller tumors on the 80×80 maps. Semantic information regarding brain tumors at varying scales is essential for accurately detecting tumor regions of different sizes. After the fusion of feature maps representing brain tumors of different scales, the FGConcat module in RRFNet is used to concatenate the semantic information. This is followed by an EMA attention mechanism that enhances the representation of spatial semantic information for brain tumors. Table 1 indicates that the performances of anchor-free brain tumor detection models surpass those of the anchor-based approaches. To further demonstrate the effectiveness of the proposed RRFNet, Fig 10 presents a comparison of the heatmaps between RRFNet and other anchor-free brain tumor detection models.

### Evaluation metrics

In object detection tasks,commonly used evaluation metrics include precision, recall, mean average precision (mAP), and frames per second (FPS). Parameters and GFLOPs reflect the complexity of the model. This section provides a detailed introduction to these metrics, which will be used to measure the performance of various comparative models in subsequent evaluations. The evaluation process involves a confusion matrix, where TP represents true positives, TN represents true negatives, FP represents false positives, and FN represents false negatives.

#### Precision

Precision measures the proportion of samples that are actually positive among those predicted as positive by the model. It focuses on the accuracy of the model’s predictions, that is, the proportion of “correctly found” cases.

$$\text{Precision}=\frac{TP}{TP+FP}$$ (11)

#### Recall

Recall measures the proportion of all actual positive samples that are correctly predicted as positive by the model. It focuses on the model’s ability to capture all relevant positive cases, representing the proportion of “completely identified” cases.

$$\text{Recall}=\frac{TP}{TP+FN}$$ (12)

#### mAP

mAP involves plotting a precision–recall (PR) curve for each class based on the values of precision and recall. This curve shows the model’s precision performance at different levels of recall. The area under the PR curve (i.e., the area beneath the curve) is the AP for that class. The higher the AP value, the better is the model’s performance on that class. For all classes, we calculate their respective AP values and then take the average of these AP values to obtain mAP.

$$mAP=\frac{1}{N}\sum_{i=1}^{N}AP_{i}$$ (13)

#### FPS

Frames per second (FPS) is a speed-related evaluation metric that represents the number of images the model can process per second. In this study, FPS is used to measure the speed at which the brain tumor detection model processes brain tumor magnetic resonance images.

$$FPS=\frac{1}{T}$$ (14)

The FPS calculation presented in this paper encompasses three components: preprocessing, inference, and postprocessing of magnetic resonance images of brain tumor.

## Experimental results

The deep learning environment for this study is based on the Ubuntu 20.04 operating system, equipped with an RTX 4090 GPU. The code is executed in an environment with CUDA 11.8 and PyTorch 2.0.0. The model is evaluated on the brain tumor validation set using the weights obtained from training, and performance metrics are calculated to assess its effectiveness. To ensure a fair comparison among different models, all model validation and inference processes are conducted on a system equipped with a 13th Gen Intel(R) Core(TM) i7-13650HX processor and an NVIDIA GeForce RTX 4060 Laptop GPU. The environment utilizes Ultralytics YOLOv8.1.19 and Python 3.9.18. When calculating the FPS, we take into account the total time spent on preprocessing, model inference, and postprocessing.

### Brain tumor detection results

Comparative experiments were conducted on Faster R-CNN, YOLOv5-anchor-free, YOLOv5-anchor based, YOLOv8, RCS-YOLO and RT-DETR to evaluate the detection performance of these models on a brain tumor dataset. The hyperparameters for training these models were kept consistent to ensure a fair comparison of their brain tumor detection performance. Specifically, the training was set for 100 epochs with a learning rate schedule of LambdaLR lrf=0.01, and an initial learning rate of 0.01 using the AdamW optimizer. Additionally, to further explore object detection models based on the DETR architecture, we have delved into two advanced object detection models, DINO and CO-DETR. In this paper, they are implemented using mmdetection [36]. The comparative results of the models are shown in Table 2. The experimental data presented in Table 2 indicate that the object detection methods not based on anchor boxes, YOLOv5 anchor-free, YOLOv8, and RT-DETR, outperformed the anchor-based object detection models, Faster R-CNN and YOLOv5. Moreover, the anchor-free design eliminated the need to set hyperparameters, such as, scale and aspect ratio, simplifying the brain tumor detection process and improving the detection speed.

**Table 2:** Experimental results of different models on the brain tumor dataset.

Model	Params(M)	GFLOPs	Precision	Recall	AP50	mAP	FPS
Faster R-CNN	41.1	134.3	83.2±0.5	85.6±0.2	92.4±0.2	65.7±0.2	-
YOLOv5n-anchor-free	2.5	7.2	96.2±0.2	94.1±0.1	97.1±0.1	76.8±0.1	91.8±2.2
YOLOv5s-anchor-free	9.1	24.0	96.2±0.2	93.6±0.3	97.2±0.1	77.3±0.2	69.9±0.5
YOLOv5m-anchor-free	25.1	64.4	95.8±0.1	93.5±0.5	97.3±0.2	76.9±0.1	58.7±2.1
YOLOv5l-anchor-free	53.2	135.3	96.1±0.5	93.7±0.1	96.6±0.3	76.5±0.1	38.5±0.5
YOLOv5x-anchor-free	97.2	246.9	96.3±0.3	92.8±0.3	96.5±0.1	76.4±0.1	21.9±0.3
YOLOv5n-anchor-base	1.8	4.2	95.2±0.5	93.3±0.2	95.2±0.2	74.8±0.2	145.1±3.1
YOLOv5s-anchor-base	7.0	16.0	95.2±0.3	93.1±0.4	94.9±0.2	75.2±0.1	126.1±4.8
YOLOv5m-anchor-base	20.9	48.2	95.8±0.4	93.3±0.2	95.7±0.3	75.3±0.2	91.5±5.5
YOLOv5l-anchor-base	46.1	108.3	95.3±0.3	93.4±0.1	95.9±0.2	75.6±0.2	54.2±0.3
YOLOv5x-anchor-base	86.2	204.7	95.6±0.2	93.0±0.2	95.2±0.3	75.1±0.2	30.6±0.5
YOLOv8n	3.0	8.2	96.2±0.2	93.8±0.4	97.2±0.2	77.6±0.2	92.8±1.0
YOLOv8s	11.1	28.7	96.3±0.2	94.1±0.2	97.3±0.3	78.2±0.3	70.1±1.5
YOLOv8m	25.9	79.1	96.5±0.1	93.9±0.3	96.7±0.2	78.3±0.2	51.8±0.5
YOLOv8l	43.6	165.4	95.2±0.3	94.0±0.2	96.5±0.4	77.4±0.4	35.4±0.3
YOLOv8x	68.2	258.1	95.9±0.3	94.2±0.2	96.7±0.3	77.7±0.2	22.4±0.3
RCS-YOLO	45.7	94.5	96.7±0.2	95.2±0.2	96.8±0.2	75.8±0.2	40.9±2.5
DINO	47.5	179.5	-	-	96.5±0.2	76.2±0.5	-
DDQ	-	-	-	-	96.1±0.4	75.8±0.3	-
RT-DETR-l	32.8	108.0	96.0±0.1	93.3±0.1	96.7±0.2	77.1±0.1	26.4±2.1
RT-DETR-x	67.3	232.3	95.6±0.2	92.9±0.1	96.3±0.2	75.8±0.4	19.9±1.2
RRFNet-n	5.3	17.8	96.5±0.3	94.9±0.2	97.5±0.1	78.3±0.2	61.7±2.2
RRFNet-s	20.6	66.8	96.3±0.2	94.6±0.3	97.6±0.3	78.5±0.2	45.6±1.2
RRFNet-m	48.3	174.2	96.5±0.2	95.1±0.1	97.8±0.2	79.2±0.3	32.5±1.6
RRFNet-l	81.8	351.2	96.2±0.1	94.8±0.1	97.5±0.1	78.8±0.1	22.4±1.6
RRFNet-x	127.8	548.2	96.7±0.2	94.9±0.2	97.3±0.2	78.6±0.1	12.8±0.7

For a fair performance comparison with other models, RRFNet was trained using the same parameter configuration as the other models listed in Table 1, including a training period of 100 epochs and a LambdaLR learning rate adjustment strategy with a decay factor set to 0.01. For the optimizer, AdamW with an initial learning rate of 0.01 was chosen.

**Fig 11:**
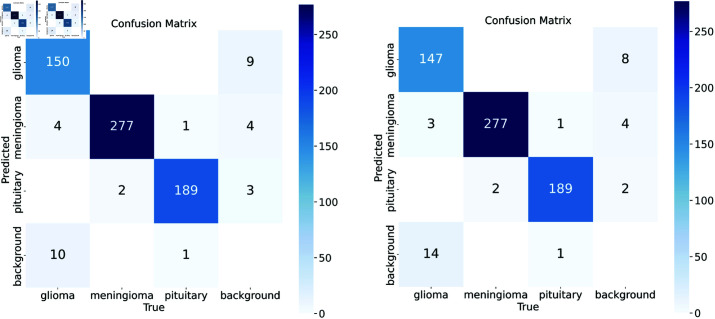
Comparison of confusion matrices. The left figure shows the results of RRFNet-m, and the right figure shows the results of YOLOv8m.

**Fig 12:**
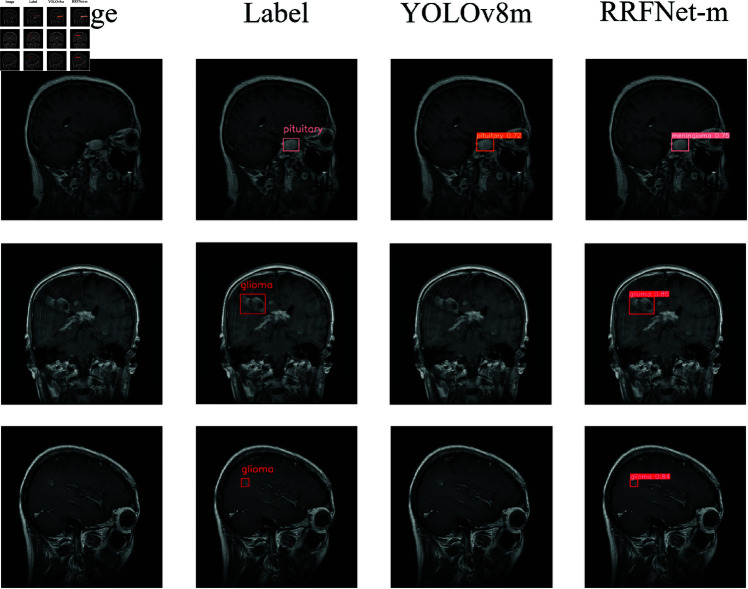
The brain tumor detection visualization comparison chart, from left to right, shows the brain tumor MRI image, the annotated brain tumor region label, and the detection visualization results of YOLOv8m and RRFNet-m.

**Fig 13:**
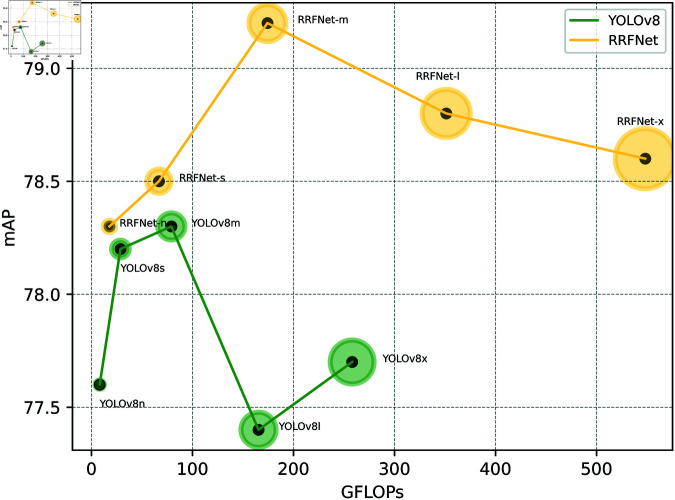
Comparison of brain tumor detection performance between RRFNet and YOLOv8.

According to the experimental results shown in Table 2, RRFNet outperformed the YOLOv8 model in terms of the mAP metric. Specifically, when comparing RRFNet-m with YOLOv8m, although RRFNet-m demonstrated an increase of 22.4M in the number of parameters and a 95.1 increase in GFLOPs, resulting in a 19.3 decrease in FPS, its mAP improved by 0.9%. This indicates that RRFNet, by introducing a reparameterized convolution module, is able to extract richer semantic information of brain tumors during the training phase, which helps the model to better understand and recognize brain tumors. To more intuitively demonstrate the performance improvement of the brain tumor detection model proposed in this paper, we compared the confusion matrices of RRFNet and YOLOv8, as shown in Fig 11, as well as the visualization results of the detection, as shown in Fig 12. It can be observed that the RRFNet model exhibits superior detection capabilities for glioma-type tumors than YOLOv8. And It has been observed that YOLOv8, while capable of detecting the general location of tumors in complex MRI images, tends to misclassify brain tumors. Moreover, YOLOv8 is prone to missing detections when the tumor tissue is similar in appearance to the surrounding normal tissue. The RRFNet model proposed in this paper effectively alleviates these problems through discriminative feature extraction and finer-grained feature fusion. During the inference phase, despite the increase in the number of parameters, RRFNet still maintains a competitive detection speed for brain tumors, striking a balance between detection performance and speed. Additionally, RRFNet effectively mitigates the issue of performance degradation with increased parameter size in the YOLOv8 model. Fig 13 illustrates the comparative performance of RRFNet and YOLOv8 in brain tumor detection. It can be concluded that RRFNet’s brain tumor detection performance is significantly better than that of YOLOv8.

### Ablation study

In this section, ablation experiments are conducted to access the effectiveness of each module in the RRFNet network. YOLOv8n is used as the baseline network, and ablation experiments are conducted on the RepConv, RepC3, and FGConcat modules. The results of the ablation experiments are presented in Table 3. The symbol “✓” means that the module is added to the baseline model; if the symbol is “-”, it means the module is not used, and instead, the default module of YOLOv8 is used as a substitute. According to the results of the ablation experiments, using FGConcat, RepConv, or RepC3 alone can enhance the model’s brain tumor detection performance; the combined use of these three modules can also improve performance. We also conducted a visual ablation study of the FGConcat module using attention map comparisons, and the results are shown in Fig 7. However, the ablation experiment results also show that while these module replacements improve performance, they increase the number of parameters and GFLOPs leading to a decrease in detection speed.

**Table 3:** Ablation study results.

RepConv	RepC3	FGConcat	Params(M)	GFLOPs	mAP
-	-	-	3.0	8.2	77.6±0.2
-	-	✓	4.2	14.7	78.0±0.2
-	✓	-	4.1	11.1	77.8±0.1
✓	-	-	3.1	8.3	77.5±0.3
✓	✓	-	4.2	11.2	77.9±0.1
✓	-	✓	4.3	14.8	77.9±0.2
-	✓	✓	5.2	17.7	77.8±0.1
✓	✓	✓	5.3	17.8	78.3±0.2

## Discussion

During the experimental process, several interesting findings were observed, offering valuable insights for subsequent brain tumor detection models.

**Impact of using anchor boxes on brain tumor detection**

Object detection methods that use anchor boxes and those that do not have their own advantages and limitations. In the experiments conducted on the brain tumor dataset in this study, it was found that avoiding the use of prior knowledge, such as anchor box scale constraints, can lead to better detection results for brain tumor models. This is because the background of brain tumors is complex, and the size of brain tumors varies considerably. If anchor boxes are used for constraints, it could lead to a decline in the accuracy of brain tumor detection and a decrease in detection speed.

**Impact of model depth and parameter volume on detection performance**

**Fig 14:**
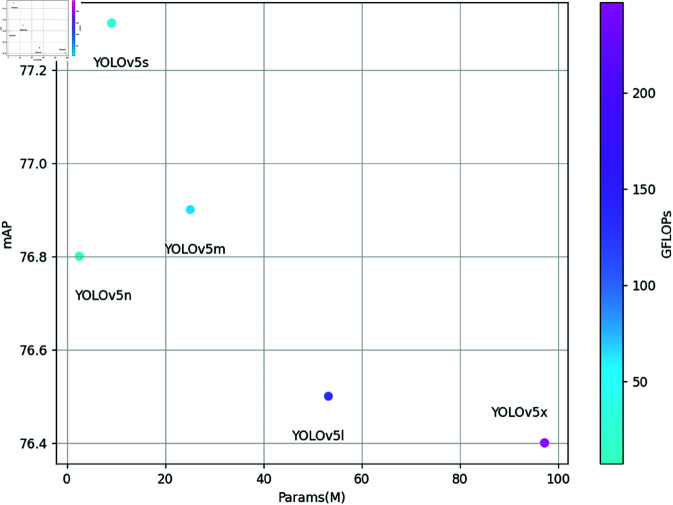
Variation chart of mAP value in brain tumor detection with parameter quantity.

In both anchor-based and anchor-free models, increasing the depth of the deep learning network initially improves detection performance. However, when the model’s parameter volume and depth become excessively large, detection performance begins to regress. Fig 14 shows changes in the performance of brain tumor detection by YOLOv5, which is an anchor-free model, as the parameter volume increases.

## Conclusion

This paper explores the impact of anchor boxes, as well as the effects of the depth and parameter volume of deep learning network models on detection performance. Based on reparameterization technology, the RRFNet model is proposed, whose backbone network extracts features more efficiently for brain tumors, providing semantic information rich feature maps for brain tumor detection. In the fusion, FGConcat is introduced to achieve more precise semantic information concatenation across different scales. The experimental results show that the proposed method significantly improved the brain tumor detection performance compared to the baseline network. Additionally, anchor-free detection models outperform anchor-based models, as the scale, aspect ratio, and other prior knowledge used in anchor boxes can limit the detection area, leading to decreased performance in complex scenarios, such as, brain tumor detection.
